# Chemical Genetics Approach Identifies Abnormal Inflorescence Meristem 1 as a Putative Target of a Novel Sulfonamide That Protects Catalase2-Deficient *Arabidopsis* against Photorespiratory Stress

**DOI:** 10.3390/cells9092026

**Published:** 2020-09-02

**Authors:** Tom van der Meer, Arno Verlee, Patrick Willems, Francis Impens, Kris Gevaert, Christa Testerink, Christian V. Stevens, Frank Van Breusegem, Pavel Kerchev

**Affiliations:** 1 VIB Center for Plant Systems Biology, B-9052 Ghent, Belgium; tom.vandermeer@wur.nl (T.v.d.M.); Patrick.Willems@psb.vib-ugent.be (P.W.); 2 Department of Plant Biotechnology and Bioinformatics, Ghent University, B-9052 Ghent, Belgium; 3 Department of Green Chemistry and Technology, Faculty of Bioscience Engineering, Ghent University, B-9000 Ghent, Belgium; arno_verlee@scarlet.be (A.V.); Chris.Stevens@UGent.be (C.V.S.); 4 VIB Proteomics Core, B-9000 Ghent, Belgium; Francis.Impens@UGent.be; 5 Department of Biomolecular Medicine, Faculty of Medicine and Health Sciences, Ghent University, B-9000 Ghent, Belgium; kris.gevaert@ugent.be; 6 VIB Center for Medical Biotechnology, B-9052 Ghent, Belgium; 7 Laboratory of Plant Physiology, Plant Sciences Group, Wageningen University and Research, 6708 PB Wageningen, The Netherlands; christa.testerink@wur.nl; 8 Department of Molecular Biology and Radiobiology, Faculty of AgriSciences, Mendel University in Brno, 61300 Brno, Czech Republic; 9 Phytophthora Research Centre, Mendel University in Brno, 61300 Brno, Czech Republic

**Keywords:** H_2_O_2_ signaling, chemical genetics, catalase2-deficient *Arabidopsis*, photorespiration, abnormal inflorescence meristem 1

## Abstract

Alterations of hydrogen peroxide (H_2_O_2_) levels have a profound impact on numerous signaling cascades orchestrating plant growth, development, and stress signaling, including programmed cell death. To expand the repertoire of known molecular mechanisms implicated in H_2_O_2_ signaling, we performed a forward chemical screen to identify small molecules that could alleviate the photorespiratory-induced cell death phenotype of *Arabidopsis* *thaliana* mutants lacking H_2_O_2_-scavenging capacity by peroxisomal catalase2. Here, we report the characterization of pakerine, an *m*-sulfamoyl benzamide from the sulfonamide family. Pakerine alleviates the cell death phenotype of *cat2* mutants exposed to photorespiration-promoting conditions and delays dark-induced senescence in wild-type *Arabidopsis* leaves. By using a combination of transcriptomics, metabolomics, and affinity purification, we identified abnormal inflorescence meristem 1 (AIM1) as a putative protein target of pakerine. AIM1 is a 3-hydroxyacyl-CoA dehydrogenase involved in fatty acid β-oxidation that contributes to jasmonic acid (JA) and salicylic acid (SA) biosynthesis. Whereas intact JA biosynthesis was not required for pakerine bioactivity, our results point toward a role for β-oxidation-dependent SA production in the execution of H_2_O_2_-mediated cell death.

## 1. Introduction

Numerous biochemical reactions involve electron transfers and are, thus, highly susceptible to alterations in the cellular redox potential. Increased levels of reactive oxygen species (ROS) have a profound impact on the molecular destiny of redox-active compounds. Whereas elevated ROS levels trigger oxidative damage, their moderate temporal and spatial fluctuations also allow for the execution of redox-based signaling relays that orchestrate growth, development, and defense [1,2]. An extensive network of enzymatic and non-enzymatic antioxidants further modulates these signaling pathways and also prevents the toxic accumulation of ROS [3]. Among the different ROS types, the relatively low reactivity and stability of H_2_O_2_ and its ability to cross membranes make it an excellent signaling molecule [4,5]. These signaling capacities are nicely demonstrated by perturbations of H_2_O_2_ levels that trigger extensive transcriptional rearrangements [6,7]. H_2_O_2_ oxidizes proteinaceous cysteinyl thiols to sulfenic acid, also known as *S*-sulfenylation, thereby affecting the protein conformation and functionality and affecting signaling pathways [8,9]. Sulfenylation and subsequent disulfide bond formation, for example, regulate jasmonic acid (JA) signaling through the inhibition of cyclophylin 20-3 (CYP20-3), the receptor of the JA precursor 12-oxophytodienoic acid (OPDA) [10,11]. H_2_O_2_-mediated cysteine oxidation in the DNA-binding motif of the transcription factor brassinazole-resistant1 (BZR1) implicated in brassinosteroid (BR) signaling enhances its transcriptional activity and promotes cell elongation [12]. Exogenous H_2_O_2_ oxidizes cysteine residues in mitogen-activated protein kinase2 (MAPK2), MAPK4, and MAPK7, activating the MAPK signaling cascade, an important signal transduction pathway in abiotic and biotic stress perception [9].

Substantial amounts of H_2_O_2_, potentially as high as 100 μM·s^−1^, are generated in the peroxisomes of photosynthetic tissues during the process of photorespiration as a byproduct of glycolate oxidation to glyoxylate by glycolate oxidase (GOX) [13]. Photorespiration is tightly intertwined with photosynthetic metabolism, and an increase in irradiance or O_2_ partial pressure inevitably results in higher rates of RuBisCO oxygenation, the initial step in the photorespiratory pathway [14]. Photorespiratory H_2_O_2_ levels are safeguarded by the enzymatic activity of catalases, and plant catalase mutants (*cat*) exposed to photorespiration-promoting conditions display a range of morphological and cell death-related phenotypes accompanied by a distinct transcriptional reprogramming and perturbation of the cellular redox homeostasis [15,16]. Catalase-deficient mutants were essential experimental model systems to identify H_2_O_2_-responsive genes and interacting signal transduction pathways, such as JA and salicylic acid (SA) signaling [17,18]. Genetic evidence for the requirement of SA accumulation for lesion formation in *Arabidopsis cat2* mutants was obtained by introducing a mutation in the SA biosynthesis enzyme isochorismate synthase1 (*sid2*) in the *cat2* background. The inability of *cat2 sid2* double mutants to increase SA levels under photorespiratory stress also abolished H_2_O_2_-induced lesion formation [19]. Similarly, impaired SA accumulation due to a mutation in the glutathione synthesis pathway (*cad2*) led to significantly reduced lesion formation under photorespiration-promoting conditions in the *cat2 cad2* double mutants [20]. Moreover, the expression of JA signaling marker genes is dependent on H_2_O_2_-induced glutathione accumulation [18]. Catalase-deficient mutants that lack the NADPH-generating cytosolic enzymes isocitrate dehydrogenase1 and 2 displayed increased lesion formation and impaired rosette growth, providing additional evidence for H_2_O_2_ signaling through glutathione since NADPH is an essential co-factor in the reduction of oxidized glutathione [21]. More than 50 genetic combinations of *cat2* with mutants affected in hormonal signaling, redox homeostasis, and cell death-related pathways were generated to systematically explore signaling cascades interacting with H_2_O_2_ [22]. Double mutant combinations with alleles defective in ascorbic acid synthesis (*cat2 vtc1* and *cat2 vtc2*) displayed decreased survival rates under conditions promoting photorespiration, whereas *cat2* mutants affected in the synthesis of the biologically active JA-isoleucine (*cat2 jar1*) and auxin signaling (*cat2 axr1 sgt1b*) performed better. The above described double and triple mutants were generated in a targeted and biased manner, thus limiting the possibility to discover conceptually new players in H_2_O_2_ signaling. Further efforts made use of forward genetics to isolate second-site mutations which can alleviate the photorespiratory phenotype of *cat2* mutants. This approach revealed a non-redundant function of the two highly similar GOX isoforms GOX1 and GOX2 in photorespiration [23]. It also led to the unbiased identification of a mutation in the transcription factor short-root (SHR), an important developmental regulator, providing a link between stress responses and plant development [24]. Together, these genetics approaches revealed the complexity of H_2_O_2_ signaling and its interplay with signal transduction pathways that regulate growth, development, and defense.

Forward genetics approaches are often limited by functional redundancy caused by gene duplication or embryo lethality for essential genes [25]. For example, the genetic dissection of the mechanism behind abscisic acid (ABA) perception failed to identify any receptor proteins. Ultimately, a chemical biology approach led to the discovery of the pyrabactin resistance (PYR/PYL) receptor proteins [26]. Small molecules can sidestep the limitations of a forward genetics approach since they can be applied in a dose-dependent manner, at selected developmental stages, and they have the potential to target multiple proteins or a protein family. For example, the small molecule bikinin inhibits seven out of the 10 *Arabidopsis* glycogen synthase kinase 3 (GSK3s), and it was used as a tool to study BR signaling [27]. Apart from their potential to deliver new biological insights, small molecules have a substantial applied potential and can be developed into agrochemicals [28].

Here, we characterized the bioactivity of pakerine, a small molecule from the *m*-sulfamoyl benzamide family that alleviates the photorespiratory-induced cell death phenotype of *Arabidopsis cat2* mutants and delays dark-induced senescence in wild-type *Arabidopsis* leaves. Using a combination of omics approaches and biochemical affinity purification, we identified the peroxisomal 3-hydroxyacyl-CoA dehydrogenase abnormal inflorescence meristem (AIM1) as its putative protein target.

## 2. Materials and Methods

### 2.1. Plant Material and Growth Conditions

*Arabidopsis thaliana* Columbia-0 (Col-0) and *Nicotiana tabacum* (SR1) were used as wild-type control plants. The following mutant lines were reported previously: *cat2-2* (SALK_057998 [15]) and *aim1-2* (SALK_023469 [29]), *cat2-1 jar1* and *cat2-1 aos* [22], and *Ntcat1* [30]. Double *cat2-2 aim1-2* were generated by crossing *cat2-*2 (pollen acceptor) with *aim1-2* (pollen donor). F2 segregating populations were screened for double mutants by PCR genotyping using respective primers (Table S1, Supplementary Materials). Wild-type PSB-L *Arabidopsis thaliana* cells (*Landsberg erecta* (*Ler*) [31]) were used as input material for the affinity purification experiments.

For photorespiratory stress treatment, seeds were surface-sterilized by chlorine gas fumigation and cold-treated at 4 °C for four days prior to germination in transparent polystyrene 96-well plates. Plants (five seeds/well) were grown in liquid half-strength Murashige and Skoog (½ MS) medium (1.07 g/L Murashige and Skoog basal salts, 0.5 g/L 2-(*N*-morpholino)ethanesulfonic acid (MES), 0.25 g/L *myo*-inositol, 0.5% *w*/*v* sucrose; pH 5.7, 200 µL/well) under controlled long-day (LD) conditions (16 h/8 h light/dark, 100 µmol·m^−2^·s^−1^ light intensity, 21 °C, 70% relative humidity). After seven days, the original medium was removed and replaced with ½ MS medium supplemented with different concentrations of pakerine (from a 20 mM stock prepared in dimethyl sulfoxide (DMSO)). Control plants received mock treatment with corresponding amounts of DMSO. Photorespiratory stress was triggered four days after media replacement by sealing the plates with four layers of Parafilm^®^ M (Bemis Flexible Packaging, USA) and exposure to continuous light conditions (24 h light, 100 µmol·m^−2^·s^−1^ light intensity, 21 °C, 70% relative humidity).

To analyze the effect of pakerine on *Arabidopsis* growth, seeds were surface-sterilized by chlorine gas fumigation and cold-treated at 4 °C for four days before germination on agar-solidified 1/2 MS medium (1.07 g/L Murashige and Skoog basal salts, 0.5 g/L 2-(*N*-morpholino)ethanesulfonic acid (MES), 0.25 g/L *myo*-inositol, 0.5% *w*/*v* sucrose, 0.8% Phyto-agar; pH 5.7) supplemented with different amounts of pakerine (0.5–50 μM). Plants were grown under LD conditions (16 h/8 h light/dark, 100 µmol·m^−2^·s^−1^ light intensity, 21 °C, 70% relative humidity). Rosette size projections were quantified using a custom-designed ImageJ macro. Root length was analyzed using the ImageJ plugin NeuronJ [32].

Dark-induced senescence was performed on detached leaves (leaves three and four) of four-week-old *Arabidopsis* seedlings grown under long-day conditions (16 h/8 h light/dark, 100 µmol·m^−2^·s^−1^ light intensity, 21 °C, 70% relative humidity). Leaves were floated, abaxial side down, in liquid 1/2 MS medium (1.07 g/L Murashige and Skoog basal salts, 0.5 g/L 2-(*N*-morpholino)ethanesulfonic acid (MES), 0.25 g/L *myo*-inositol, 0.5% *w*/*v* sucrose; pH 5.7) supplemented with pakerine or DMSO. Leaves were placed in the dark for seven days at 21 °C to provoke dark-induced senescence.

### 2.2. Chlorophyll Fluorescence

Photosystem II (PSII) maximum efficiency (F_v_’/F_m_’) was monitored daily using the Imaging-PAM MAXI-series chlorophyll fluorescence system (PAM) (Heinz Walz, Effeltrich, Germany. F_v_’ is calculated by subtracting the baseline fluorescence (F_o_’) emitted by PSII, from the maximum fluorescence (F_v_’) emitted from PSII after applying a saturated pulse of actinic light (wavelength 450 nm, intensity 2800 μmol·m^−2^·s^−1^, time 1 s). F_v_’/F_m_’ is a calculated as (F_m_’ − F_o_’)/F_m_’. The F_v_’/F_m_’ parameter is visualized spatially with Imaging-Win software using a false color rainbow scale ranging from black (F_v_’/F_m_’ = 0) via red, yellow, orange, green, and blue to purple (F_v_’/F_m_’ = 1).

### 2.3. Metabolite Profiling

Wild-type *Arabidopsis* seedlings were grown vertically on 1/2 MS medium supplemented with 1 μM pakerine or DMSO under controlled long-day conditions (16 h/8 h light/dark). Three weeks post germination, whole seedlings were harvested, freeze-dried, and processed at Metabolon (https://www.metabolon.com) using proprietary methods for sample extraction and gas chromatography (GC)/liquid chromatography (LC)–mass spectrometry (MS) analysis. Briefly, samples were prepared using Hamilton MicroLab STAR^®^ liquid handling system, divided into two fractions, and the organic solvent was removed on a TurboVap^®^ (Zymark). Samples were then frozen and dried under vacuum. The samples for GC–MS analysis were re-dried under vacuum desiccation for a minimum of 24 h prior to being derivatized under dried nitrogen using bistrimethyl-silyl-triflouroacetamide (BSTFA). A 16-min temperature gradient, ramping from 40 to 300 °C, on a 5% phenyl column was used. The samples were analyzed on a Thermo-Finnigan Trace DSQ fast-scanning single-quadrupole mass spectrometer using electron impact (EI) ionization. For LC–MS analysis, the sample extract was split into two aliquots, dried, and then reconstituted in acidic or basic LC-compatible solvents, containing injections standards at fixed concentrations. The corresponding aliquots were analyzed in positive and negative ionization mode using separate dedicated columns. The acidified aliquots were eluted using water and methanol both containing 0.1% HCOOH. The basic aliquots were eluted with water/methanol containing 6.5 mM NH_4_HCO_3_. The samples were analyzed on Waters ACQUITY UPLC and a Thermo-Finnigan LTQ mass spectrometer, which consisted of an electrospray ionization (ESI) source and linear ion-trap (LIT) mass analyzer. The MS analysis alternated between MS and data-dependent MS2 scans using dynamic exclusion. Raw data were extracted, peak-identified, and QC processed. Compounds were identified by comparison to library entries of purified standards using retention index, nominal mass (±0.4 amu), and MS/MS.

### 2.4. Transcriptome Profiling

#### 2.4.1. ATH1 Microarray Analysis

*Arabidopsis* Col-0 seedlings were grown in liquid MS medium (0.2% sucrose, pH 5.7) in a 24-well plate set up under constant environmental conditions (16 h/8 h light/dark, 100 μmol·m^−2^·s^−1^ light intensity, 22 °C). After seven days, the original medium was replaced with medium supplemented with 5 μM pakerine (20 mM stock in DMSO). Control plants received mock treatment with corresponding amounts of DMSO. After 24 h, seedlings from individual wells were pooled to obtain three replicates for the treated and control group, snap-frozen in liquid nitrogen, and used to extract RNA with the RNeasy Plant Mini Kit (Qiagen). Gene expression profiles were analyzed using Affymetrix *Arabidopsis* ATH1 genome arrays following standard protocols. Quality control and robust multi-array average (RMA) normalization were performed using the R package *affy*. Fold-change differences and *p*-values adjusted for false discovery rate were obtained with the R packages *limma* and *qvalue*, respectively. A cut-off of *p*-value ≤ 0.05 and log 2 expression ratio ± 1 was adopted. The gene expression data were deposited in Gene Expression Omnibus (GEO; http://www.ncbi.nlm.nih.gov/geo/) under accession number GSE155026.

#### 2.4.2. RNA Sequencing

*Arabidopsis cat2* seedlings were grown in liquid ½ MS medium for seven days in LD conditions, after which the original medium was replaced with medium containing 10 μM pakerine or equal amounts of DMSO. Non-stressed plants were harvested after four days of incubation in LD conditions. Photorespiratory stressed plants were harvested after an additional two days of photorespiratory stress. Three biological replicates per treatment were collected (100 mg leaf material/replicate), snap-frozen, and ground to a fine powder. Total RNA was isolated using the Spectrum^TM^ Plant Total RNA Kit (Sigma-Aldrich, St. Louis, MO, USA). Library preparation and sequencing was performed by the VIB Nucleomics Core (www.nucleomics.be). The samples were analyzed on an Illumina NextSeq 500. In total, approximately 30 million 75-bp single-end reads per sample were generated. Adapter sequences and low-quality base pairs (length below 20 bp) were trimmed with Trim Galore v0.3.3 (http://www.bioinformatics.babraham.ac.uk/projects/trim_galore/), retaining high-quality reads of at least 50 bp in length. Quality-filtered reads were aligned to the TAIR10 *Arabidopsis* reference genome using the spliced aligner TopHat2 v2.1.0. The number of reads per gene was quantified with the feature “Counts” function as implemented in the Subread package v1.4.6. Reads mapping to genes annotated as ribosomal RNA (rRNA), transfer RNA (tRNA), and other RNA forms (TAIR10 annotation) were not considered for further analysis. Differentially expressed genes were identified with the R v3.1.2 software package edgeR [33]. Genes with expression values greater than 0.12 CPM (count per million, corresponding to five read counts) in at least three samples were retained. TMMnormalization was applied using the calcNormFactors function. Variability in the dataset was assessed with a MDSplot employing the 3000 top genes to calculate pairwise distances. False discovery rate adjustments of the *p*-values were determined with the method described by Benjamini and Hochberg. Venn diagrams were made using an online tool (http://bioinformatics.psb.ugent.be/webtools/Venn/). Hierarchical clustering and heat maps were generated using MultiExperiment Viewer v4.9. Gene ontology (GO) enrichment analysis was performed using AgriGO (http://bioinfo.cau.edu.cn/agriGO/). The gene expression data were deposited in Gene Expression Omnibus (GEO; http://www.ncbi.nlm.nih.gov/geo/) under accession number GSE155105.

### 2.5. Structure–Activity Relationship Analysis

Pakerine analogues were synthesized according to the method described by Verlee et al. [34]. Briefly, a continuous-flow method was developed to increase the chemoselectivity and reduced production time allowing for quicker production of chemical libraries with higher purity. *Cat2* plants were grown in liquid cultures in 96-well plates as described above and treated with different concentrations of the analogues (0.1–150 μM). PSII maximum efficiency (F_v_’/F_m_’) was monitored daily using the Walz Imaging-PAM system from the moment the chemicals were administered to the plants.

### 2.6. Affinity Purification

*A. thaliana* PSB-L cell suspension cultures were grown until log-phase (approximately three days) before harvesting and frozen at −70 °C. After grinding to powder (500 mg), proteins were extracted from cells in extraction buffer (EB) (25 mM Tris-HCl pH 7.5, 150 mM NaCl, 0.1% NP-40, 1 Complete Ultra Mini EDTA-free tablet (Roche)/10 mL EB) in a 1:2 *w*/*v* ratio. All steps were carried out on ice or at 4 °C unless stated otherwise. After vigorous mixing, the suspension was incubated on ice for 25 min. Cell debris was removed by centrifuging at 20,200 rcf for 25 min. Supernatant was transferred to a new tube and centrifuged for an additional 15 min at 20,200 rcf. Next, protein content was determined by Bradford assay. Protein content of approximately 3 mg/mL was used for the experiment. Streptavidin-coated beads were incubated with cell lysate to remove endogenous biotin. Then, 100 µL of Streptavidin-coated beads were washed three times with 500 µL of wash buffer (WB) (25 mM Tris-HCl pH 7.5, 150 mM NaCl, 0.1% NP-40) by inverting them several times followed by a spin down of 1 min at 1400 rcf. A fourth washing step was done with 500 µL of EB. The washed beads were incubated with 500 µL of cell lysate for 2 h on a rotating wheel. After 2 h, the beads were removed by spinning the cell lysate at 1400 rcf for 1 min. The cell lysate was subsequently diluted two-fold with EB. Two 100-µL aliquots of Streptavidin beads were first washed in a similar fashion to that described above: three washes with WB followed by one wash with EB. Beads were resuspended in 500 µL of EB. To one aliquot, the biotinylated pakerine analog SA-004 (BSA-004) was added to a final concentration of 50 µM. To the second aliquot, Biotin-PEG (BPEG) was added to a final concentration of 50 µM, which served as a control treatment. Both mixes were incubated for 1 h at room temperature on a rotating wheel. After 1 h, the supernatant was removed after centrifugation (1 min, 1400 rcf, 4 °C). Then, 500 µL of the de-biotinylated cell lysate was mixed with each aliquot of the streptavidin-capturing beads and incubated for 2 h at room temperature on a rotating wheel. After 2 h, the mix was transferred to mobicol affinity chromatography columns (MoBiTec GmbH, Göttingen, Germany) for washing and elution from streptavidin beads. Streptavidin beads were washed with 2.5 mL of EB. Next, the beads were washed with 800 µL of 50 mM NH_4_HCO_3_ pH 8.0. After removal of all the wash buffer, 200 µL of 50 mM NH_4_HCO_3_ pH 8.0 and 4 µL (20 ng/µL) of Trypsin/LysC were added to the beads and incubated overnight in an Eppendorf shaker at 37 °C, 600 rpm. After overnight trypsin digest, an additional 2 µL (20 ng/µL) of Trypsin/LysC (Promega, Madison, WI, USA) was added, and the beads were incubated for two more hours in the shaker at 37 °C, 600 rpm. Finally, the beads were spun down, and the flow-through containing the digested peptides was collected. Peptides were eluted from the columns by two washes with 150 µL of mass spec-grade water. The flow-throughs per sample were collected and combined. Finally, the samples were dried in the speedvac and stored at −20 °C until further mass spectrometry analysis.

### 2.7. Mass Spectrometry and Data Analysis

The obtained peptide mixtures were analyzed using a Q Exactive mass spectrometer (Thermo Fisher Scientific, Bremen, Germany) connected to an Ultimate 3000 RSLC nano liquid chromatography system (Thermo Fisher Scientific). The sample mixture was first loaded on a trapping column (made in-house, 100 μm internal diameter (I.D.) × 20 mm, 5-μm beads C18 Reprosil-HD, Dr. Maisch, Ammerbuch-Entringen, Germany). After flushing from the trapping column, the sample was loaded on an analytical column (made in-house, 75 μm I.D. × 150 mm, 5 μm beads C18 Reprosil-HD, Dr. Maisch) packed in the needle (PicoFrit SELF/P PicoTip emitter, PF360-75-15-N-5, New Objective, Woburn, MA, USA). Peptides were loaded with loading solvent (0.1% trifluoroacetic acid (TFA) in water) and separated with a linear gradient from 98% solvent A’ (0.1% formic acid in water) to 40% solvent B′ (0.1% formic acid in water/acetonitrile, 20/80 (*v*/*v*)) in 30 min at a flow rate of 300 nL/min. This was followed by a 5 min wash reaching 99% solvent B’. Two packing columns and two analytical columns were configured in tandem LC mode, and switching between two flow paths—an analysis flow path and a regeneration flow path—allowed column washing and re-equilibration off-line; thus, while one column was re-equilibrated, the system injected a sample on the other column. The mass spectrometer was operated in data-dependent, positive ionization mode, automatically switching between MS and MS/MS acquisition for the 10 most abundant peaks in a given MS spectrum. The source voltage was 3.4 kV, and the capillary temperature was 275 °C. One MS1 scan (*m*/*z* 400–2000, AGC target 3 × 10^6^ ions, maximum ion injection time 80 ms) acquired at a resolution of 70,000 (at 200 *m*/*z*) was followed by up to 10 tandem MS scans (resolution 17,500 at 200 *m*/*z*) of the most intense ions fulfilling predefined selection criteria (AGC target 5 × 10^4^ ions, maximum ion injection time 60 ms, isolation window 2 Da, fixed first mass 140 *m*/*z*, spectrum data type: centroid, underfill ratio 2%, intensity threshold 1.7 × 10^4^, exclusion of unassigned, 1, 5–8, >8 charged precursors, peptide match preferred, exclude isotopes on, dynamic exclusion time 20 s). The HCD collision energy was set to 25% normalized collision energy, and the polydimethylcyclosiloxane background ion at 445.120025 Da was used for internal calibration (lock mass).

Raw data files from the LC–MS/MS analysis were processed using MaxQuant (ver. 1.5.7.0), followed by statistical analysis in Perseus (ver. 1.5.6.0). MaxQuant parameters used for analysis can be found in Table S2 (Supplementary Materials).

## 3. Results

### 3.1. Two m-Sulfamoyl Benzamides Alleviate the Photorespiratory Phenotype of Arabidopsis cat2-2 Mutants

**Figure 1:**
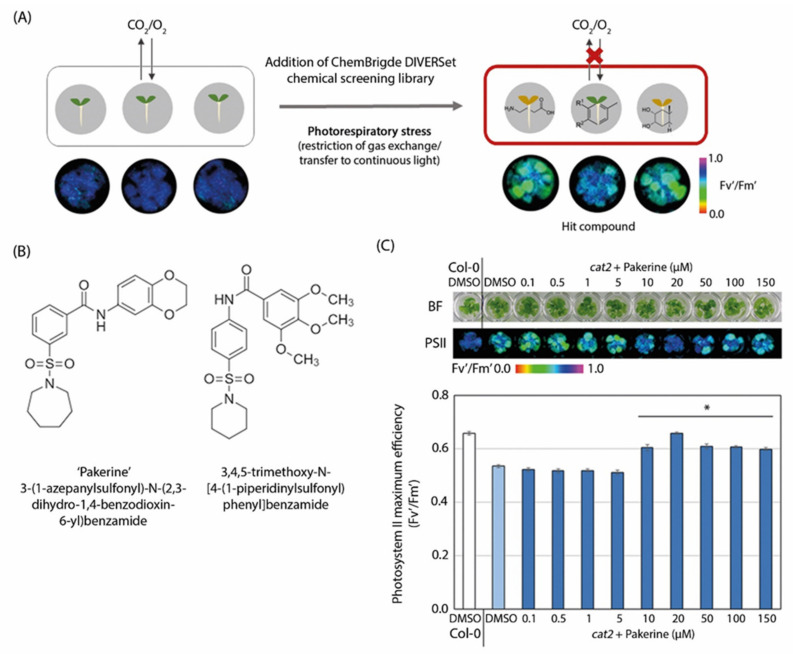
Strategy to identify small molecules that alleviate the photorespiratory phenotype of catalase-deficient plants together with chemical structures of two hit compounds. (**A**) Schematic depiction of the forward chemical screed used to isolate small molecules that attenuate photosystem II (PSII) maximum efficiency (F_v_’/F_m_’) decrease in *cat2-2* mutants plants exposed to photorespiratory stress (sealing plates airtight with Parafilm and transfer to continuous light). In total, 10,000 chemicals from the DIVERSet screening library were tested in 50 µM concentration. Shown are representative color-coded F_v_’/F_m_’ images (black (0.0) to purple (1.0)) before and after chemical addition and exposure to photorespiratory stress. (**B**) Chemical structures of two of the hit compounds identified in the forward chemical screen. (**C**) Effect of different concentrations of pakerine on the photorespiratory phenotype of *cat2-2* mutant plants. Bright-field (BF) and color-coded F_v_’/F_m_’ (PSII) images (upper panel) together with quantitative F_v_’/F_m_’ data (lower panel) from seven-day-old seedlings treated with pakerine and exposed to photorespiratory stress for seven days. Control plants were treated with dimethyl sulfoxide (DMSO). Data points represent means of eight biological replicates ± standard error (SE). Asterisks indicate significant differences according to one-way ANOVA with least significant difference (LSD) post hoc test (*p* < 0.05).

In a previously reported forward chemical screen, we identified 34 small molecules that could alleviate the cell death phenotype of *cat2-2* mutants under photorespiration-promoting conditions [35]. Briefly, one-week-old *cat2-2* seedlings grown in a 96-well plate set-up were screened with 10,000 molecules (50 µM) from the ChemBridge’s DIVERSet chemical library under photorespiratory stress. Photorespiratory stress was imposed by restricting gas exchange between the plate and the environment with exposure to continuous light (Figure 1A). This combination gradually depletes the CO_2_ levels within the plate, thereby leading to increased rates of RuBisCO oxygenation that fuels the photorespiratory pathway [24,36]. Plants lacking catalase (*cat2-2*) cannot adequately process photorespiratory H_2_O_2_ and display a cell death phenotype within seven days of the onset of photorespiratory stress. We used the chlorophyll fluorescence parameter photosystem II maximum efficiency (F_v_’/F_m_’) as a proxy for stress sensitivity. Typically, F_v_’/F_m_’ decreases rapidly in *cat2-2* mutant plants exposed to photorespiratory stress compared to wild-type plants that display relatively stable F_v_’/F_m_’ values over time. Small molecules that could alleviate the stress sensitivity caused by the photorespiratory stress and led to the attenuation of cell death were retained as hit compounds (Figure 1A) [35].

Two of these shared a characteristic *m*-sulfamoyl-benzamide moiety, suggesting a common mode of action (Figure 1B). Due to its more advantageous chemical properties for synthesis of structural analogues, we prioritized 3-(1-azepanylsulfonyl)-*N*-(2,3-dihydro-1,4-benzodioxin-6-yl)benzamide (further referred to as pakerine) for subsequent analysis. First, we assessed the ability of pakerine to alleviate photorespiratory stress in a dose-dependent manner. Therefore, *cat2-2* seedlings were treated with a range of pakerine concentrations (100 nM–150 µM) and exposed to photorespiratory stress. While no stress-alleviating effects were detected below 10 µM, concentrations from 10 µM to 150 µM significantly attenuated the F_v_’/F_m_’ decline observed in mock-treated *cat2-2* control plants exposed to photorespiratory stress (Figure 1C).

To investigate whether the stress-protective effect of pakerine can be observed outside the context of the model system *Arabidopsis*, we treated tobacco (*Nicotiana tabacum*) plants lacking catalase1 (*Ntcat1*), the homolog of *Arabidopsis CAT2* [30]. *Ntcat1* mutants displayed a comparable F_v_’/F_m_’ decline upon exposure to photorespiratory stress as *Arabidopsis cat2-2* mutant plants (Figure S1, Supplementary Materials). Pakerine at concentrations from 5 µM to 150 µM alleviated the F_v_’/F_m_’ drop observed in *Ntcat1* mutants under conditions promoting photorespiration suggesting that pakerine’s mode of action is similar in different plant species.

### 3.2. Pakerine Treatment Results in Distinct Transcriptome Rearrangement upon Photorespiratory Stress

**Figure 2:**
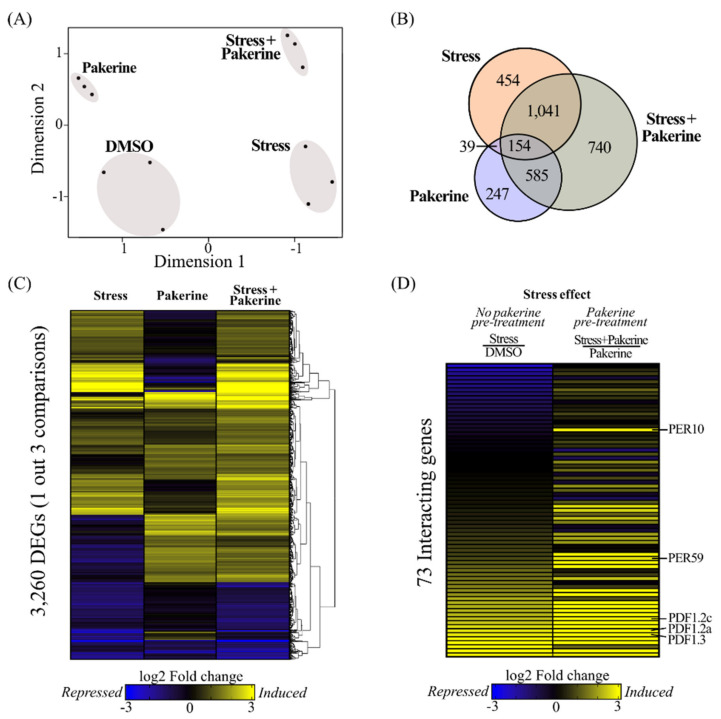
Rearrangement of gene expression in *cat2-2* plants upon photorespiratory stress in the presence of pakerine. (**A**) Multidimensional scaling plot of transcriptome profiles of *cat2-2* mutant plants treated with pakerine or DMSO before and after exposure to photorespiratory stress. “DMSO”: DMSO-treated plants under control conditions; “Pakerine”: pakerine-treated plants under control conditions; “Stress + Pakerine”: pakerine-treated plants exposed to photorespiratory stress; “Stress”: DMSO-treated plants exposed to photorespiratory stress. (**B**) Venn diagram depicting the overlap between differentially expressed genes (DEGs; |Log_2_ fold change (FC)| > 1, false discovery rate (FDR) < 0.01) in plants exposed to photorespiratory stress (“Stress”), pakerine-treated plants (“Pakerine”), and pakerine-treated plants exposed to photorespiratory stress (“Stress + Pakerine”) against mock-treated plants (“DMSO”). (**C**) Hierarchical average clustering of 3260 DEGs identified in at least one out of the three pairwise comparisons described in Figure 2B. (**D**) Heat map of 73 transcripts that responded to photorespiratory stress in a pakerine-specific manner according to two-way (pakerine × photorespiratory stress) ANOVA.

To probe for molecular pathways underpinning the stress alleviation effect of pakerine, we monitored the transcriptome in mock and pakerine-treated (10 µM) *cat2-2* plants, before and after exposure to 48 h of photorespiratory stress. In a multidimensional scaling plot, differentially expressed genes were clearly separated into four discrete clusters according to pakerine treatment (*y*-axis) and photorespiratory stress (*x*-axis), indicating that both conditions have a specific effect on global gene expression (Figure 2A).

Photorespiratory stress altered the expression levels of 1688 genes in mock-treated *cat2-2* plants, whereas pakerine affected the levels of 1025 genes under control conditions (|log_2_ fold change (FC)| ≥ 1; false discovery rate (FDR) ≤ 0.01; Figure 2B; Table S3, Supplementary Materials). Photorespiratory stress in pakerine pretreated plants showed a dramatic transcriptome reprogramming with 2520 differentially expressed genes (Figure 2B). The effect of photorespiratory stress mostly masked the pakerine-induced transcriptional changes (Figure 2C). Nevertheless, 73 transcripts responded to the photorespiratory stress in a pakerine-specific manner according to two-way (pakerine × photorespiratory stress) ANOVA (Figure 2D; Table S4, Supplementary Materials). Genes that were highly induced upon photorespiratory stress in the presence of pakerine but showed no upregulation in pakerine-treated non-stressed plants were the peroxidases PER10 (*At1g49570*) and PER59 (*At5g19890*). In contrast, three transcripts encoding for plant defensins (*PDF1.2a*, *PDF1.2c*, *PDF1.3)* were significantly downregulated under non-stressed conditions but increased upon pakerine treatment during photorespiratory stress. Taken together, the transcriptional changes elicited by pakerine point toward subtle but distinct transcriptional reprogramming correlating with its stress-alleviating effects.

### 3.3. Bioactivity of Pakerine in Wild-Type Arabidopsis

**Figure 3:**
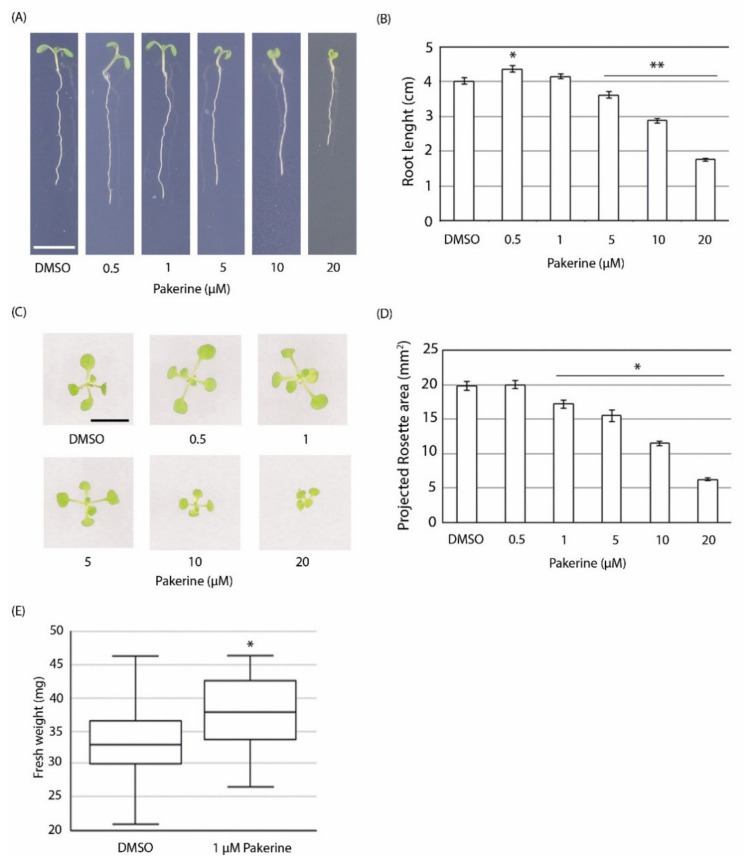
Effect of pakerine on the growth of wild-type *Arabidopsis*. (**A**) Representative images of seven-day-old seedlings grown vertically on MS medium supplemented with different pakerine concentrations. Control plants were grown on medium supplemented with DMSO. Scale bar = 1 cm. (**B**) Quantification of the primary root length of seedlings (*n* = 36) grown on pakerine as described in (**A**). Asterisks depict significantly longer (*) or shorter (**) roots compared to DMSO control according to one-way ANOVA with LSD post hoc test (*p* < 0.05). Error bars represent SE. (**C**) Representative images of rosettes of 11-day-old plants grown on half MS medium supplemented with different pakerine concentrations. Scale bar = 0.5 cm. (**D**) Quantification of the projected rosette area of plants (*n* = 36) grown as described in (**C**). Asterisks depict significant differences according to one-way ANOVA with LSD post hoc test (*p* < 0.05). Error bars represent SE. (**E**) Biomass of three-week-old plants grown on half MS medium supplemented with 1 μM pakerine. Control plants were grown on medium supplemented with DMSO. Box plots represent data from 32 different observations. Asterisks indicate significant differences according to Student’s *t*-test (*p* < 0.05).

We then set out to assess the bioactivity of pakerine outside of the photorespiratory bioassay in the *cat2-2* mutant model system. To this end, wild-type *Arabidopsis* seedlings were grown on agar-solidified medium that was supplemented with a range of pakerine concentrations, and their effect on root and shoot growth was evaluated (Figure 3).

Low pakerine concentrations had a subtle yet statistically significant growth-promoting effect, resulting in an increase of root length by 8.4% at 0.5 µM pakerine (A,B). A trend toward an increase in projected rosette area was similarly observed at low pakerine concentrations (Figure 3C,D) which prompted us to explore the effect of pakerine on plant growth in a longer time frame. Three-week-old plants grown on medium supplemented with 1 µM pakerine displayed increased biomass (15%) in comparison to pakerine-free controls (Figure 3C,E). In contrast, the growth of plants exposed to higher pakerine concentrations (10 µM and 20 µM) was significantly inhibited already at seven days post germination (Figure 3A–D).

**Figure 4:**
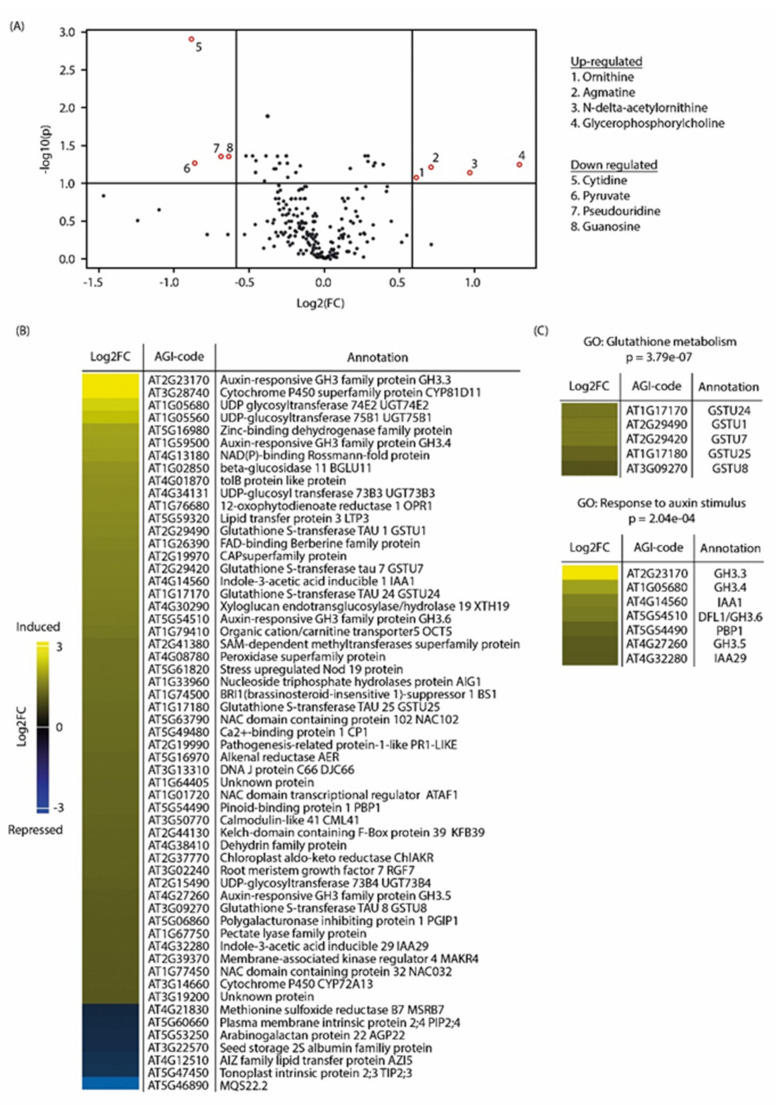
Transcriptome and metabolite changes triggered by pakerine in wild-type *Arabidopsis*. (**A**) Differentially regulated metabolites (|FC| ≥ 1.5; *p* ≤ 0.05) extracted from three-week-old plants grown in the presence of 1 μM pakerine. Controls were exposed to DMSO. (**B**) Heat map of differentially expressed transcripts (|log2 FC| ≥ 1; adjusted *p* ≤ 0.05) in 10-day-old *Arabidopsis* seedlings treated with 5 μM pakerine for 24 h. Controls received mock treatment with DMSO. (**C**) Gene ontology (GO) analysis of differentially expressed transcripts depicted in (**B**). Shown are two significantly enriched clusters (*p* < 0.05).

To explore the mechanisms that underlie the growth-promoting effect provoked by a low dose of pakerine, we first performed a metabolite profiling analysis of three-week-old *Arabidopsis* wild-type plants grown on medium supplemented with 1 µM pakerine (Figure 4A, Table S5, Supplementary Materials).

The steady-state levels of more than 250 metabolites were quantified using gas and liquid chromatography coupled to mass spectrometry (GC/LC–MS). Pakerine treatment resulted in up- or downregulation of eight metabolites in comparison to mock samples (|FC| > 1.5, *p* < 0.05; Figure 4A). Among the metabolites that accumulated in the presence of pakerine was the polyamine ornithine, its closely related metabolite *N*^5^-acetyl-l-ornithine, and agmatine, a precursor of putrescine biosynthesis.

We then monitored the transcriptional changes triggered by a short-term pakerine treatment (5 µM; 24 h). From the 58 differentially regulated transcripts (|log_2_ FC| ≥ 1; adjusted *p* ≤ 0.05), 52 were induced by pakerine (Figure 4B; Table S6, Supplementary Materials). Among the upregulated genes were several H_2_O_2_ responsive transcripts, such as *UGT74E2*, *ANAC032*, *ANAC102,* and *At4g01870* (tolB protein-like). Gene ontology analysis distinguished two enriched clusters containing transcripts related to glutathione metabolism (*p* = 3.8 × 10^−7^) and auxin homeostasis (*p* = 2.0 × 10^−4^; Figure 4C). Using the Signature tool in Genevestigator, we looked for experimental conditions that provoked similar gene expression patterns [37]. The top three most similar signatures based on Pearson correlation were related to hypoxia and pathogenic fungal (*Rhizoctonia solani*) infection (Figure S2, Supplementary Materials). Taken together, these results indicate that pakerine displays a wider bioactivity that is not limited to the photorespiratory bioassay in *cat2-2* mutant background and has a broad impact on transcripts and metabolites known to be involved in stress signaling cascades.

### 3.4. Pakerine Alleviates Dark-Induced Senescence

**Figure 5:**
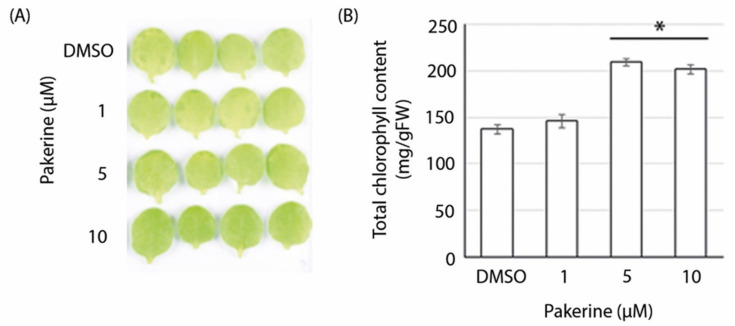
Pakerine delays dark-induced senescence in wild-type *Arabidopsis* leaves. (**A**) Representative images of detached leaves incubated on liquid MS media supplemented with pakerine or DMSO for seven days in darkness. (**B**) Total chlorophyll content of leaves treated as in (**A**). Data points represent means of 12 individual leaves ± SE. Asterisks indicate significant differences according to one-way ANOVA with LSD post hoc test (*p* < 0.05).

Senescence and programmed cell death in plants share many common features, although the exact transcriptional and metabolic profiles that distinguish their initiation are not clearly defined [38]. Dark-induced senescence (DIS) in detached leaves is widely accepted as an experimental system to study senescence symptoms, such as chlorophyll degradation and protein catabolism, also typically observed during abiotic stress [39]. Given the similarities between photorespiratory-induced cell death phenotypes in *cat2-2* mutant plants and DIS, we investigated whether pakerine can also alleviate DIS. Leaves from three-week-old *Arabidopsis* plants grown in vitro were floated in darkness on medium supplemented with different pakerine concentrations (1, 5, and 10 µM). Leaves incubated with 5 and 10 µM pakerine were visually greener and retained a higher total chlorophyll content (52% and 47%, respectively) after seven days, demonstrating that pakerine indeed can alleviate the effects of DIS in wild-type plants (Figure 5).

### 3.5. Structure–Activity Relationship (SAR) Analysis of Pakerine

**Figure 6:**
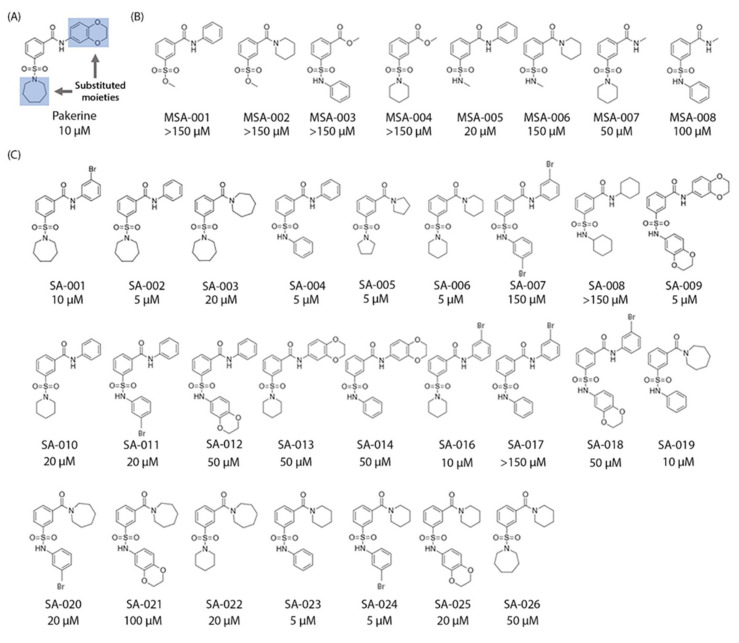
Structure–activity relationship (SAR) analysis of pakerine. (**A**) Original chemical structure of pakerine. Functional groups that were modified in the synthesized analogues are highlighted in blue. (**B**) Analogues with single substitution in the original structure. (**C**) Analogues with two substitutions in the original structure. The active concentrations at which the analogues attenuated F_v_’/F_m_’ decline in *cat2-2* mutants exposed to photorespiratory stress are shown below each structure.

Affinity purification, using a small molecule as bait, can discover its protein targets. Therefore. functionalized analogues, entailing an affinity tag, allow target enrichment within a complex protein mixture [40]. In order to design a bioactive functionalized pakerine analogue, we first assessed which functional groups are required for the stress-alleviating effects of pakerine through a SAR analysis. A series of 33 pakerine analogues was synthesized and subsequently tested in a broad concentration range (100 nM–150 µM) for their ability to alleviate the photorespiratory phenotype of *cat2-2* mutant plants. The structure of pakerine was altered by substituting the heterocyclic and aromatic moieties attached to the amide and/or sulfonamide moieties (Figure 6A).

**Figure 7:**
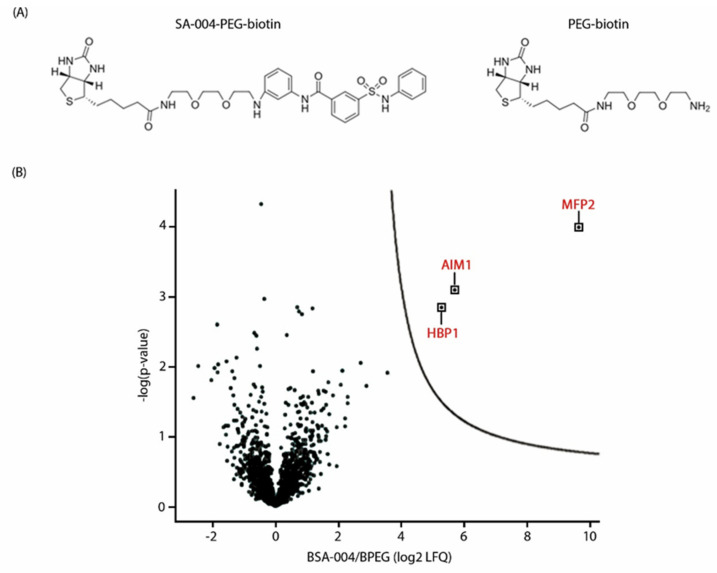
Abnormal inflorescence meristem 1 (AIM1) is a putative protein target of pakerine. (**A**) Chemical structures used for affinity purification. The functionalized pakerine analogue SA004 fused to a polyethylene glycol (PEG) linker and coupled to a biotin tag at the end of the PEG chain (SA004–PEG–Biotin) was used to fish out putative protein interactions. A construct consisting of biotin attached to PEG (Biotin–PEG) was used to eliminated background proteins interacting with the matrix and the linker. (**B**) Proteins significantly enriched after affinity purification. Protein isolates from *Arabidopsis* cell cultures (*n* = 3) were incubated with SA004–PEG–Biotin and significantly enriched proteins in comparison to Biotin–PEG controls were identified (log_2_ label-free quantification intensity (LFQ) > 3.5, *p* < 0.05).

Replacement of the heterocyclic or aromatic moieties from either the amide or the sulfonamide moiety, with the simultaneous removal of opposite heterocyclic or aromatic moiety, leaving a free amide or sulfonamide moiety, resulted in eight analogues (Figure 6B). Replacement of the sulfonamide moiety in MSA-001 and MSA-002 by sulfonic acid and substitution of the amide moiety in MSA-003 and MSA-004 by an ester group led to a complete loss of activity. Removal of the aromatic ring from the sulfonamide side in MSA-005 and MSA-006 and of the heterocyclic moiety from the amide side in MSA-007 and MSA-008 also resulted in reduced bioactivity. Next, 25 analogues with different heterocyclic and aromatic ring structures on both the amide and sulfonamide side were synthesized to identify favorable conformations for the ring structures that might lead to improved bioactivity. The structural analogues SA-002, SA-004, SA-005, SA-006, SA-009, SA-023, and SA-024 displayed increased bioactivity in comparison to pakerine and were able to alleviate the photorespiratory phenotype of *cat2-2* mutant plants at 5 µM concentration (Figure 6C; Table S7, Supplementary Materials). SA-004 was functionalized by linkage of a biotinylated polyethylene glycol moiety to its benzene ring (SA-004–PEG–biotin; Figure 7A).

### 3.6. The Peroxisomal Enzyme Abnormal Inflorescence Meristem 1 (AIM1) Is a Putative Target of Pakerine and Is Necessary for Pakerine-Induced Alleviation of Dark-Induced Senescence

*Arabidopsis* protein extracts were subjected to affinity purification using SA-004–PEG–biotin and PEG–biotin (negative control) as baits (Figure 7A). Three proteins were significantly enriched in the SA-004–PEG–biotin versus the PEG–biotin control binding fraction (Figure 7B). The peroxisomal enzyme abnormal inflorescence meristems 1 (AIM1; At4g29010), which is a 3-hydroxyacyl-CoA dehydrogenase involved in fatty acid β-oxidation, was exclusively trapped by SA-004–PEG–biotin, but not by control PEG–biotin. Noteworthy is that the two other enriched proteins (5.7- and 9.6-fold, respectively) heme-binding protein 1 (HBP1; At1g17100) and multifunctional protein 2 (MFP2; At3g06860) were also detected in the PEG-biotin control binding fraction (Table S8, Supplementary Materials). Interestingly, MFP2 is highly similar to AIM1 (75% amino-acid sequence similarity) and also acts in the peroxisomal β-oxidation pathway, implying that it can be targeted by pakerine. AIM1 was prioritized for functional characterization since it was exclusively trapped in the SA-004–PEG–biotin binding fraction.

To obtain genetic evidence for the involvement of AIM1 in the modulation of the photorespiratory phenotype of *cat2-2* mutant plants, we set out to generate *aim1-2 cat2-2* double mutants. However, double *aim1-2 cat2-2* homozygous mutant seeds were non-viable, as approximately 23% of the mature F3 seeds (18–19 days after fertilization) were aborted, suggesting that the combined effect of *cat2-2* and *aim1-2* results in seed lethality (Figure S3, Supplementary Materials) [41].

**Figure 8:**
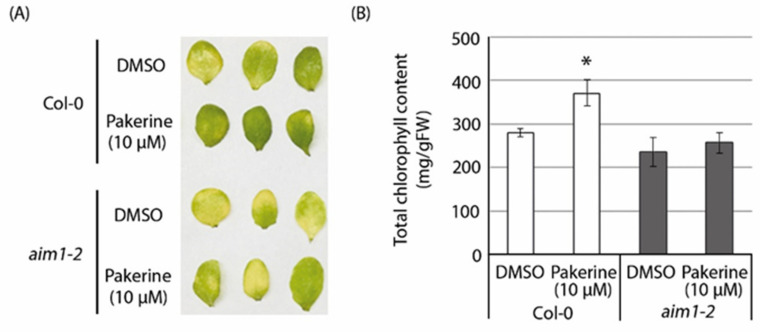
Pakerine does not alleviate dark-induced senescence in the absence of AIM1. (**A**) Representative images of detached *aim1-2* and wild-type *Arabidopsis* leaves incubated on liquid MS medium supplemented with pakerine (10 μM) or DMSO (control) for seven days in darkness. (**B**) Total chlorophyll content of leaves treated as in (**A**). Data points represent means of 12 individual leaves ± SE. Asterisks indicate significant differences according to one-way ANOVA with LSD post hoc test (*p* < 0.05).

We then assessed how the absence of AIM1 affects dark-induced senescence. Detached leaves of three-week-old *aim1-2* mutants displayed accelerated senescence in comparison to the wild type (Figure 8A,B). Interestingly, pakerine (10 µM) did not affect the senescence of *aim1-2* mutant leaves.

### 3.7. JA Signaling Is Not Required for Pakerine Activity

**Figure 9:**
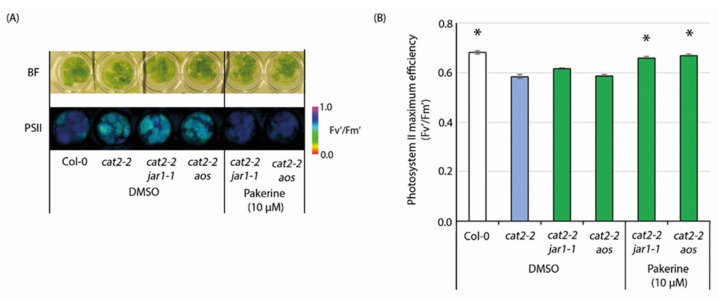
Jasmonic acid (JA) signaling is not required for pakerine function. (**A**) Representative bright-field (BF) and color-coded F_v_’/F_m_’ (PSII) images of *cat2-2 jar1-1* and *cat2-2 aos* double mutants treated with 10 μM pakerine and exposed to photorespiratory stress together with mock-treated controls. (**B**) Quantitative F_v_’/F_m_’ data from plants treated as in (**A**). Data points represent means of eight individual measurements ± SE. Asterisks indicate significant differences according to one-way ANOVA with LSD post hoc test (*p* < 0.05).

AIM1 acts in the final steps of JA biosynthesis and converts 12-oxophytoenoic acid-CoA into 7-*iso*-JA, the precursor of the bioactive jasmonoyl-isoleucine (JA-Ile) conjugate [42]. The *aim1* mutants show dramatic developmental defects and are severely affected in wound-induced JA accumulation and expression of JA-responsive genes [43]. Interestingly, the expression of three JA-responsive genes *PDF1.2a*, *PDF1.2c*, and *PDF1.3* was repressed by pakerine in *cat2-2* mutants under control conditions but still induced upon exposure to photorespiratory stress (Figure 2D). To further investigate the interplay between JA signaling and pakerine activity, we assessed whether JA biosynthesis is required for pakerine-mediated alleviation of the photorespiratory phenotype of *cat2-2* mutants. To this end, we exposed *aos cat2-1* and *jar1 cat2-1* double mutants, which are unable to synthesize the JA precursor 12-oxophytodienoic acid (OPDA) and JA-Ile, respectively, to photorespiratory stress in the presence of pakerine. Both *aos cat2* and *jar1 cat2* double mutants displayed a drop in F_v_’/F_m_’ upon exposure to photorespiratory stress comparable to that of single *cat2* mutants (Figure 9).

Addition of pakerine (10 µM) still counteracted the F_v_’/F_m_’ decline in all genotypes, indicating that intact JA signaling is not required for pakerine function.

## 4. Discussion

We characterized the sulfonamide 3-(1-azepanylsulfonyl)-*N*-(2,3-dihydro-1,4-benzodioxin-6-yl) benzamide, dubbed pakerine, that could alleviate the photorespiratory-dependent cell death phenotype of catalase-deficient *Arabidopsis* and tobacco plants. Through affinity-based chemoproteomics, we isolated the peroxisomal 3-hydroxyacyl-CoA dehydrogenase AIM1 as a putative target of pakerine in plants.

Many sulfonamides are bioactive and have applications as antibiotics in mammals and as safeners in plants [44]. The safeners metcamifen and cyprosulfonamide used in monocot crops, for example, induce the expression of cytochrome P450s and glutathione transferases, which likely catalyze the detoxification of the herbicide clodinafop-propargyl [45,46]. Quinabactin, which activates the ABA receptors PYR1, PYL 1–3, and PYL5 and confers drought tolerance in *Arabidopsis* and soybean, also contains a sulfonamide group [47,48]. The *m*-sulfamoyl benzamide group of pakerine was essential for its ability to alleviate the photorespiratory phenotype of *cat2-2* mutant plants, which classifies pakerine as a novel bioactive sulfonamide. Pakerine was not only active in the photorespiratory bioassay, but also affected the root length and shoot size of wild-type *Arabidopsis* seedlings and delayed dark-induced senescence. Interestingly, low pakerine concentrations (1 μM) promoted growth, whereas growth inhibition was observed under high concentrations. Such biphasic responses are typically associated with hormesis, which describes the stimulation of many independent cellular processes resulting in a beneficial effect at moderate stress levels as opposed to a high dose-inhibitory effect [49]. In contrast to the clear dose–response inhibition of root and rosette growth by pakerine, no linear dose–response effect was observed when a range of pakerine concentrations were tested for their potency to alleviate the photorespiratory phenotype of *cat2-2* mutant plants. These variations can stem among others from differences in pakerine uptake and distribution in roots and shoots or distinct protein targets that mediate both physiological effects.

### 4.1. Abnormal Inflorescence Meristem1 Is a Putative Protein Target of Pakerine

Attaching biotinylated polyethylene glycol needed to fish out interacting proteins inevitably affects the pharmacokinetics of the bait molecule. This functionalization rendered the construct SA-004–PEG–biotin, which was used for affinity purification, inactive in contrast to the functional pakerine analogue SA-004, most likely due to a reduced uptake and inability to reach its protein target (Figure S4, Supplementary Materials). We used protein lysates from *Arabidopsis* cell cultures to sidestep the requirement for uptake of SA-004–PEG–biotin. Apart from AIM1, which was exclusively found in the SA-004–PEG–biotin binding fraction, two additional proteins that were significantly enriched in the SA-004–PEG–biotin binding fraction were also trapped by the PEG–biotin control. One of these, multifunctional protein 2 (MFP2), shares high amino-acid sequence similarity (75%) with AIM1. Both MFP2 and AIM1 are peroxisomal enzymes involved in fatty-acid β-oxidation that possess 2-*trans*-enoyl-CoA hydratase and l-3-hydroxyacyl-CoA dehydrogenase activities [50]. Despite their high sequence similarity and overlapping enzymatic activities, however, MFP2 and AIM1 play distinct roles in plant development, which suggests that pakerine might exert its activity by targeting only one of them. Under long-day conditions, mutants lacking AIM1 are dwarfed and display impaired flower development and reduced seed set [51]. In contrast, mutants lacking MFP2 do not display any growth defects [52]. The *aim1* seeds are resistant to indole-3-butyric acid (IBA) and 2,4-diphenoxybutyric acid (2,4-DB), indicating perturbation of peroxisomal function, since IBA and 2,4-DB are activated to indole acetic acid (IAA) and 2,4-dichlorophenoxyacetic acid (2,4-D), respectively, through a series of β-oxidation steps [51,53]. Oppositely, mutants lacking MFP2 are susceptible to IBA and 2,4-DB [52,54]. Both *aim1* and *mfp2* cannot germinate without an external carbon source, and *aim1 mfp2* double mutants do not produce viable seeds [52].

Leaf senescence is characterized by increased expression of enzymes involved in fatty-acid β-oxidation supporting the breakdown of long-chain fatty acids from membrane degradation [55]. Mutants lacking 3-ketoacyl-CoA thiolase 2 (KAT2), an essential enzyme in the peroxisomal β-oxidation pathway, show delayed natural and dark-induced senescence [56]. In contrast, *pxa1* mutants affected in peroxisomal ABC transporter 1, involved in uptake of fatty acyl-CoAs, display accelerated dark-induced senescence [57]. Whereas pakerine delayed dark-induced senescence in the wild type, detached *aim1* leaves senesced faster and were not affected by the presence of pakerine. Even though it is tempting to equate pharmacological inhibition of a protein to a genetic mutation, in many cases, the resulting phenotypes are different, which might explain the observed senescence phenotypes [58]. On the other hand, genes involved in auxin homeostasis were overrepresented upon pakerine treatment in the wild type. The available evidence points toward an active role of auxin in senescence accompanied by changes in auxin biosynthesis and signaling [59]. The *auxin/indole-3-acetic acid* (*AUX/IAA*) genes, which are negative regulators of auxin responses, are downregulated in senescing leaves, and exogenous auxin transiently decreases the expression of the senescence marker *SAG21* [60]. Moreover, a pro-auxin, which was discovered in the same chemical screen as pakerine, was able to alleviate the cell death phenotype of catalase-deficient mutants exposed to photorespiratory stress [35]. Pakerine induced the expression of IAA29, which is downregulated in senescing leaves, implying that the delayed dark-induced senescence can be at least partially related to pakerine’s impact on auxin homeostasis. Given that the endogenous precursor of auxin indole 3-butyric acid (IBA) is converted into the active indole 3-acetic acid (IAA) in the peroxisomes, perturbations of fatty-acid β-oxidation might impact auxin homeostasis [61]. Further supporting the impact of pakerine on senescence-related pathways was the accumulation of the polyamine ornithine, its closely related metabolite *N*^5^-acetyl-l-ornithine, and agmatine, a precursor of putrescine biosynthesis. Polyamines were shown to delay senescence and protect plants against various environmental stresses [62,63].

### 4.2. Pakerine Acts Independently of JA Signaling

Peroxisomal oxidation plays an important role in hormonal homeostasis. The synthesis of auxin, JA, and SA involves peroxisomal β-oxidation steps mediated by fatty-acid β-oxidation enzymes, including AIM1 and MFP2. The JA precursor OPDA is first reduced to 12-oxophytoenoic acid, which, after three successive rounds of β-oxidation, is converted into 7-*iso*-JA [42]. The involvement of JA in the regulation of cell death was reported under various adverse environmental conditions [64]. For example, *Arabidopsis* plants exposed to excess light and heat stress showed a dramatic increase in OPDA, JA, and the bioactive JA conjugate JA-Ile, which was accompanied by increased cell death and decreased survival rates [65]. On the other hand, *allene oxide synthase* (*aos*) mutants impaired in OPDA biosynthesis displayed decreased survival rates [65]. Overexpression of wheat *allene oxide cyclase (AOC)* leads to increased OPDA and JA biosynthesis, which is accompanied by improved fitness under salinity stress [66]. Interestingly, *aim1* mutants are unable to mount wound-induced JA synthesis and JA-responsive gene expression [43]. The effect of pakerine on JA-related gene expression suggested that it might exert its effect via modulation of JA homeostasis likely through inhibition of the enzymatic activity of AIM1 involved in JA synthesis. Previously, *cat2* mutants that carry a second-site mutation in *jasmonate resistant1* (*JAR1*) and are unable to synthesize the bioactive JA conjugate JA-Ile displayed reduced lesion formation and increased survival rate under photorespiratory stress [22]. In our photorespiratory bioassay, however, *cat2-2 jar1* double mutants phenocopied *cat2-2* single mutants, indicating that the lack of bioactive JA does not influence the stress response. This apparent discrepancy can be attributed to differences in growth conditions, such as light period (12-h light regime versus continuous light in our experiments), which are important determinants of cell death in catalase-deficient mutant plants [15]. To further refute the role of JA signaling in pakerine function, *cat2-2 jar1* and *cat2-2 aos* double mutants, deficient in the JA-precursor OPDA and having lower overall JA levels, showed similar pakerine-triggered Fv’/Fm’ attenuation in the photorespiratory assay as single *cat2-2* mutants.

### 4.3. SA Accumulation Is a Likely Player in Pakerine Function

AIM1 is involved in the biosynthesis of secondary metabolites derived from benzoic acid (BA), such as benzoylated glucosinolates and substituted hydroxybenzoylcholines, which are important players in biotic stress interactions [29]. As a result, *aim1* mutants display significantly lower levels of BA-derived metabolites, including SA, which is partially synthesized via the phenylalanine ammonia-lyase (PAL) pathway [29]. The AIM1 enzymatic activity responsible for SA synthesis is likely the conversion of *trans*-cinnamic acid into BA. A rice mutant lacking AIM1 displayed 30% lower SA levels in roots than the wild type, which was accompanied by a decrease in BA content and accumulation of its precursor *trans*-cinnamic acid [67]. Interestingly, this mutant showed reduced root meristem activity and repressed ROS levels, likely stemming from increased expression of redox and ROS-scavenging-related genes. One plausible explanation of the protective role of pakerine is, hence, through the modulation of H_2_O_2_ homeostasis. Pakerine had no apparent effect of the cellular redox homeostasis; however, given the significant impact of the photorespiratory stress and the intrinsic difficulties associated with quantifying H_2_O_2_ levels, pakerine’s impact on H_2_O_2_ might not be easily identifiable. SA accumulation dependent on isochorismate synthase 1 (ICS1) is necessary for lesion formation in catalase-deficient *Arabidopsis* mutants under photorespiratory conditions [19]. ICS1 functions in the isochorismate (ICS) pathway, which is the predominate pathway for biotic stress-inducible SA biosynthesis [68]. The importance of ICS and PAL pathways varies between plant species and is process-dependent. Whereas SA biosynthesis in tobacco seems to occur primarily through the PAL pathway, both the ICS and the PAL pathways are equally important in pathogen-triggered SA biosynthesis in soybean [69,70]. The involvement of PAL-derived SA in the photorespiratory phenotype of *cat2-2* was not previously shown. Impaired SA accumulation due to perturbation of the peroxisomal β-oxidation step in SA biosynthesis might similarly alleviate the negative effects of photorespiratory stress and deserves further investigation.

In this study, we characterized a novel sulfonamide that could alleviate the negative effects of photorespiration in catalase-deficient mutants and delayed dark-induced senescence. Both phenotypes could be explained by pakerine inhibition of the peroxisomal enzyme AIM1 involved in fatty-acid β-oxidation, which was purified as a putative target of pakerine. Taken together, our results indicate a role of fatty-acid β-oxidation in H_2_O_2_-induced cell death.

## Supplementary Materials

The following are available online at https://www.mdpi.com/2073-4409/9/9/2026/s1: Figure S1. Reversion of photorespiratory-induced F_v_’/F_m_’ decline in *Nicotiana tabacum cat1* mutants by pakerine; Figure S2. Perturbations showing similarity to pakerine-induced transcriptional changes in wild-type *Arabidopsis*; Figure S3. Impaired seed development in *aim1-2 cat2-2* double mutant plants; Figure S4. Photorespiratory stress alleviating effect of the chemical probes used for affinity purification; Table S1. Primer sequences used for genotyping of T-DNA insertion lines; Table S2. MaxQuant parameters used for mass spectrometry data analysis; Table S3. Global transcriptome profiling of *cat2-2* plants treated with pakerine under control and photorespiratory stress; Table S4. Transcripts that responded to the photorespiratory stress in a pakerine-specific manner according to two-way ANOVA; Table S5. Metabolite profiling of wild-type plants grown in the presence of pakerine; Table S6. Pakerine-induced gene expression in wild-type *Arabidopsis*; Table S7. Structure–activity relationship analysis of 34 pakerine analogues; Table S8. List of identified proteins after affinity purification.
